# Adjuvant Chemotherapy, a Valuable Alternative Option in Selected Patients with Cervical Cancer

**DOI:** 10.1371/journal.pone.0073837

**Published:** 2013-09-13

**Authors:** Shuang Li, Ting Hu, Yile Chen, Hang Zhou, Xiong Li, Xiaodong Cheng, Ru Yang, Shixuan Wang, Xing Xie, Ding Ma

**Affiliations:** 1 Department of Obstetrics and Gynecology, Tongji Hospital, Tongji Medical College, Huazhong University of Science and Technology, Wuhan, P.R. China; 2 Department of Gynecologic Neoplasms, Hunan Province Tumor Hospital, The Affiliated Tumor Hospital of Central South University, Changsha, People’s Republic of China; 3 Women’s Reproductive Health Laboratory of Zhejiang Province, Zhejiang, P.R. China; Kinghorn Cancer Centre, Garvan Institute of Medical Research, Australia

## Abstract

Radiotherapy is the standard treatment for cervical cancer, but causes radiotherapy-induced complications. Recently, chemotherapy has been more extensively utilized. Here, we perform a large-scale comparison of chemotherapy and radiotherapy. From 2002 to 2008, 2,268 patients were grouped according to adjuvant radiotherapy or chemotherapy before and/or after surgery, and we compared the 5-year overall survival (OS) and disease-free survival (DFS) rates, recurrence rates, side effects, quality of life (QoL), and sexual activity. There were no significant differences between the treatment groups for the 5-year OS and DFS rates (OS: *p = *0.053, DFS: *p* = 0.095), although marginally improved outcomes were observed in the chemotherapy group (OS: 86.5% vs. 82.8%; DFS: 84.5% vs. 81.4%). However, patients with early-stage disease, clinical response, and younger age had increased 5-year OS and DFS rates following chemotherapy compared to radiotherapy (*p*<0.05). The chemotherapy group exhibited significantly lower 5-year recurrence and distant failure rates compared to the radiotherapy group (*p*<0.001 and *p* = 0.007, respectively). Nausea and vomiting were the most frequent short-term complications of chemotherapy, whereas bowel and urinary complications were more frequent in the radiotherapy group. Compared to the chemotherapy group, patients who received radiotherapy reported a lower QoL, less frequent sexual activity, and more severe menopausal symptoms (*p*<0.05). Cervical cancer patients treated with chemotherapy, especially those with early-stage disease, clinical responses, and younger ages, have more positive outcomes, fewer complications, better QoL and sexual activity, suggesting that chemotherapy may be a valuable alternative option for selected patients.

## Introduction

Cervical cancer is the second most common cancer in women from developing countries and is a leading cause of cancer-related death in women worldwide [1]. Several studies have documented a steady increase in the incidence of early-stage cervical cancer incidence and cervical cancer in young women in several counties [2]–[5]. For decades, cervical cancer has been most commonly treated with radical surgery and radiotherapy, which are listed as the standard treatments in the clinical practice guidelines [6], [7]. Five-year survival rates of 74%–91% have been reported for patients with stage IB-IIA cervical cancer after radiotherapy; these rates are similar to those for radical surgery (83%–91%) [8]. Several recent studies have demonstrated a survival advantage with the use of concurrent chemoradiotherapy, making this treatment a generally accepted alternative option for patients with stage IB2 and worse diseases in many counties [9], [10].

The current cervical cancer treatment outcomes are acceptable; however, critical reviews have described the prevalence of long-term survivors suffering from somatization and menopausal symptoms, depression, and sexual dysfunction after radiotherapy [11]–[15]. Patients who receive radiotherapy and concurrent chemoradiotherapy may also experience early small bowel obstruction, early or late large bowel complications (bleeding, stricture, fistulae), late urinary complications (hematuria, ureteral stenosis), or vaginal atrophy, shortening, or agglutination; the latter can make sexual intercourse difficult or even impossible [9], [16]. Furthermore, the ovaries are permanently destroyed by a radiation dose above 6∼10 Gy, even when an ovary transposition is performed. It is important to consider the possibility of organ damage and the loss of female endocrine ability, particularly in cervical cancer patients who are diagnosed at younger ages and have a high chance of being cured due to early-stage disease. These younger and possibly nulliparous women may expect to live an additional 20–30 years after treatment and may be eager to preserve fertility [15].

Chemotherapeutic strategies have recently become a dominant treatment for malignant gynecological tumors. Gestational trophoblastic tumors are highly susceptible to chemotherapy, and a high cure rate can often be achieved while preserving reproductive function [17]. The outcomes in patients with malignant ovarian germ cell cancers largely depend on sensitivity to chemotherapy [18]. The 2009 International Federation of Gynecology and Obstetrics (FIGO) clinical practice guidelines recommend neoadjuvant chemotherapy for selected patients with stage IB2-IIB cervical cancer [7], and clinical practice confirms that adjuvant chemotherapy significantly suppresses distant metastasis in patients with intermediate and high risk factors [19]. These findings suggest that it is worth re-examining the value of chemotherapy modalities for selected patients, in terms of survival benefits and simultaneously improved quality of life (QoL).

Although the FIGO and the National Comprehensive Cancer Network (NCCN) have not recommended chemotherapy as a conventional primary treatment for cervical cancer, increasing numbers of patients with pathological risk factors have been treated with chemotherapy after surgery in the past ten years. Here, we performed a large-scale comparison of the treatment efficacy, complications, QoL and sexual activity in patients with stages IB-IIIB cervical cancer who were treated with either adjuvant chemotherapy or radiotherapy, to provide clinicians and patients with empirical information to help guide their treatment choices.

## Patients and Methods

### Patients

From 2002 to 2008, 2,268 eligible cases were collected from the cervical cancer database v1.10 (10,897 patients; http://clinicaltrials.gov; Figure S1). This study protocol was approved by the Ethics Committee of Tongji hospital, Tongji Medical College, Huazhong University of Science and Technology, P. R. China. The Ethics Committee of Tongji hospital, Tongji Medical College, Huazhong University of Science and Technology approved the Waiver of Consent Requirements for this research (No. 20120303).

The inclusion criteria were as follows: patients with FIGO stage IB-IIIB cervical cancer (squamous, adeno-, or adenosquamous and small cell carcinoma); patients treated with radical hysterectomy and bilateral pelvic lymphadenectomy; patients who received either chemotherapy or radiotherapy, but not both; and patients with no existing complicating diseases or prior malignant diseases. Adjuvant treatments were incorporated by neo-adjuvant (before surgery) treatment and post-surgery treatment. The chemotherapy group consisted of patients treated with adjuvant chemotherapy before and/or after surgery, and the radiotherapy group consisted of patients treated with adjuvant radiotherapy before and/or after surgery. Cervical cancer patients with risk features including low grade, adenocarcinoma or adenosquamous (AC/ASC) histology, or bulky disease (>4 cm), etc. were treated with the preoperative adjuvant chemotherapy or radiotherapy. Patients who underwent primary radical hysterectomy and were found to have some risk factors for recurrence, including LNM, positive resection margins, parametrial invasion, large tumor size, lymphovascular space invasion and >1/3 stromal invasion, AC/ASC histology, or low grade, etc. were treated with the postoperative adjuvant chemotherapy or radiotherapy. Patients were excluded from the study if they met any of the following criteria: medical contraindications for chemotherapy or radiotherapy, patients who received both chemotherapy and radiotherapy; patients who received concurrent chemoradiotherapy; and patients with rare histological subtypes, prior malignant diseases, or other conditions that might not permit completion of the study and the required follow-up. High risk factors (HRFs) included LNM, positive resection margins, and parametrial invasion [20].

Data were assembled using a standardized data form on patient demographics, history, clinical presentations, physical and gynecological examinations, colposcopy, cervical biopsy, routine full blood count, renal and hepatic function tests, imaging study results, and details of surgical and adjuvant treatments. HPV status is not known for the partial patients when the patient data were collected.

### Treatments

Cisplatin-based chemotherapy modalities were employed before and/or after surgery ( Table S1) [21]. Adjuvant chemotherapy was administered in 1–2 courses before surgery or 2–6 courses after surgery, depending on tolerance and response. In accordance with the World Health Organization criteria, the clinical response to chemotherapy was evaluated at the initial diagnostic procedure and before surgery [22]. Clinical responders were defined as patients with a complete response (CR) or partial response (PR), and non-responders included patients with stable disease (SD) or progressive disease (PD) [20], [21]. Adjuvant radiotherapy consisted of external beam irradiation and intracavitary brachytherapy before surgery or within 4–6 weeks postoperatively (Table S2). The requirements for continuing full-dose adjuvant therapy included a white blood cell count ≥ 3,000/mm^3^, a platelet count ≥ 100,000 and a serum creatinine level ≤ 2.0 mg/dl.

### Side Effects

Short-term side effects were defined as those occurring during treatment or within 90 days of completing therapy. Long-term side effects occurred more than 90 days after completing therapy. Treatment-related toxicity was assessed at the time of each evaluation, according to the National Cancer Institute Common Toxicity Criteria, version 3.0 [23].

### QoL and Sexual Activity Assessment

The QoL and sexual activity were assessed in patients who were <50 years old with unilateral or bilateral ovary preservation, using the European Organization for Research and Treatment of Cancer Quality of Life Core Questionnaire (EORTC QLQ-C30) [24], the Cervix Cancer Module (QLQ-CX24) [25], seven additional questions about menopausal symptoms[26], and the Sexual Activity Questionnaire (SAQ) [27].

### Follow-up

Patients were suggested to be re-evaluated every three months during the first year, and every six months during the subsequent four years. The results of these examinations and any changes in therapy, adverse effects, or disease progression, as well as any deaths were reported.

### Statistical Analysis

Statistical analyses were performed with SPSS 13.0 software package. *p<*0.05 was considered significant. Summary statistics are presented as frequencies and percentages (mean ± SD). The Pearson’s chi-squared or Fisher’s exact tests were used for categorical data. Disease-free survival (DFS) and overall survival (OS) were estimated using the Kaplan-Meier analysis for different groups. The log-rank test was used to compare survival curves. Sites of recurrence were classified as local if detected in the pelvis or vagina, and distant if detected in extrapelvic locations. T-tests were used to compare EORTC QLQ-C30, QLQ-CX24, and SAQ scores between the chemotherapy and radiotherapy groups, whereas the Pearson’s chi-squared test was used to compare the scores of menopausal symptoms.

## Results

### Patient Characteristics

The 2,268 eligible patients were stratified into two groups (Figure S1). There were no significant differences in age (*p* = 0.058) or marriage status (*p* = 0.132, Table S3) of the patients in the two groups. The patients in the chemotherapy group (1,010 patients) had more serious disease characteristics than the patients in the radiotherapy group (1,258 patients), including larger tumor size, AC/ASC histological subtypes, and later stages disease stages (Table S3). The median follow-up time was 41 months (range; 4–108 months) in this study.

### Outcomes

**Figure 1:**
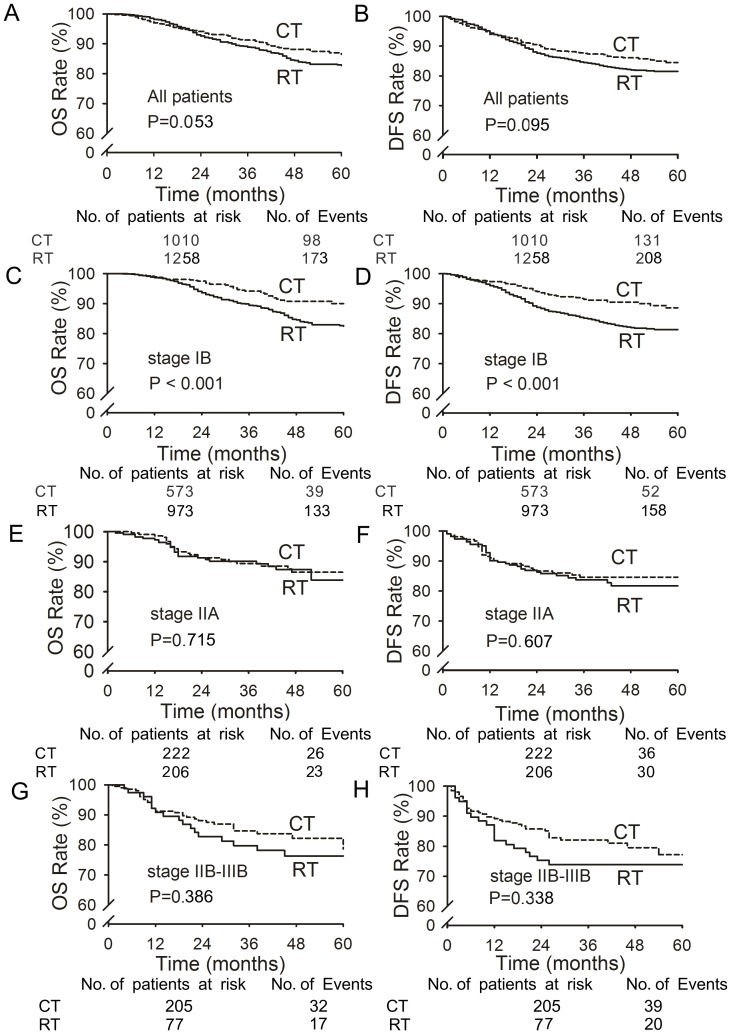
Overall survival (OS) and disease-free survival (DFS) of patients in the chemotherapy (CT) and radiotherapy (RT) groups. Panels A and E show all patients (1,010 CT and 1,258 RT). Panels B and F show patients with stage IB cervical cancer (573 CT and 973 RT). Panels C and G show patients with stage IIA (222 CT and 206 RT). Panels D and H show patients with stage IIB-IIIB (205 CT and 77 RT).

**Figure 2:**
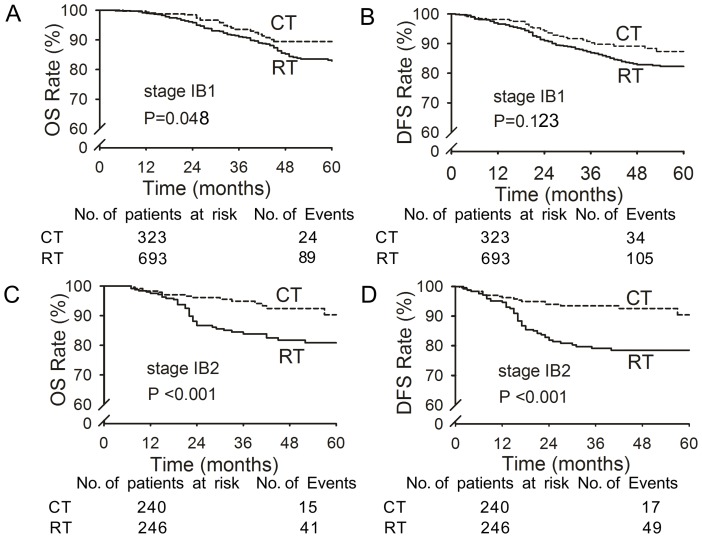
Overall survival (OS) and disease-free survival (DFS) of patients with stage IB1 and IB2 cervical cancer in the chemotherapy (CT) and radiotherapy (RT) groups. Panels A and C show OS and Panels B and D show DFS of patients with stage IB1 (323 CT and 693 RT) or IB2 (240 CT and 246 RT) cervical cancer.

The 5-year OS and DFS rates were slightly but not significantly higher in the chemotherapy group compared with the radiotherapy group (OS: 86.5% vs. 82.8%, *p* = 0.053, Fig. 1A; DFS: 84.5% vs. 81.4%, *p* = 0.095, Fig. 1B). Among patients with stage IB cervical cancer, the 5-year OS and DFS rates were significantly higher in the chemotherapy group than the radiotherapy group (*p*<0.001 for OS and DFS; Fig. 1C and 1D). There were no significant differences between the 5-year OS and DFS rates in patients with stages IIA and IIB-III (*p*>0.05; Figs. 1E–1H); furthermore, within this subgroup, the differences between treatment groups were larger in patients with stage IB2 disease (OS: *p*<0.001; DFS: *p*<0.001; Figs. 2C and 2D) and were substantially smaller in patients with stage 1B1 disease (OS: *p* = 0.048, Fig. 2A; DFS: *p* = 0.123, Fig. 2B).

**Figure 3:**
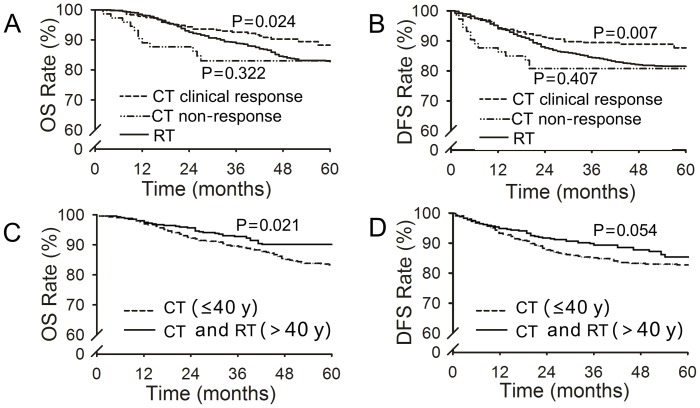
Overall survival (OS) and disease-free survival (DFS) of patients treated with chemotherapy (CT) or radiotherapy (RT), stratified by clinical response and age. Panels A and B show OS and DFS in the CT with (*N* = 471) or without (*N* = 73) clinical response groups and the RT group (*N* = 1,258). Panels C and D show OS and DFS of patients ≤40 years in the CT group (*N* = 418) and all patients aged >40 years in the CT and RT groups (*N* = 1,370).

Stratifying the analysis according to clinical response to chemotherapy revealed more details about survival. In the chemotherapy clinical response group, the 5-year OS and DFS rates were significantly increased compared to those in the radiotherapy and chemotherapy clinical non-response groups (OS: *p* = 0.024, Fig. 3A; DFS: *p* = 0.007, Fig. 3B). In the chemotherapy non-clinical response group, the 2-year OS and DFS rates were significantly decreased compared to those in the radiotherapy and chemotherapy clinical response groups; however, the 5-year OS and DFS rates were not significantly different from those in the radiotherapy group (OS: *p* = 0.322, Fig. 3A; DFS: *p* = 0.407, Fig. 3B).

Next, we stratified patients by age. Patients ≤40 years old in the chemotherapy group exhibited increased 5-year OS and DFS rates compared to patients who were >40 years old (OS: *p* = 0.021, DFS: *p* = 0.054, Figs. 3C and 3D). We also analyzed the survival data for patients with HRF, and found no significant differences in the 5-year OS and DFS rates between patients treated with chemotherapy and those treated with radiotherapy, when examining either all patients or patients divided into different disease stage groups (Fig. S2; *p*>0.05).

### Recurrence

At 5 years, 282 of 1,258 women (22.4%) developed a cancer recurrence after radiotherapy, including 180 (14.3%) with local (vagina or pelvis) recurrences and 139 (11.1%) with distant metastases (Table 1). In contrast, only 167 (16.5%) cases of recurrence, including 98 (9.7%) with local recurrence and 78 (7.7%) with distant recurrence, occurred among the 1,010 patients who underwent chemotherapy (*p*<0.001). At 5 years, patients in the chemotherapy group exhibited significantly lower local recurrence and distant failure rates compared to those in the radiotherapy group (*p*<0.001 and *p* = 0.007); no significant difference in distant failure rates was found at 2 years (*p* = 0.071). The most frequent distant recurrence sites were lung (23 patients, 2.3%) and bone (23 patients, 2.3%) after chemotherapy, and lung (57 patients, 4.5%), bone (36 patients, 2.9%), and distant lymph nodes (34 patients, 2.7%) after radiotherapy.

**Table 1:** Comparison of Recurrence Rates and Sites, and Side Effects between the Chemotherapy and Radiotherapy Groups.

	Radiotherapy Group (*N = *1,258)	Chemotherapy Group (*N = *1,010)		*p* value
	*no. of patients (%)*			
Recurrence rates and sites				
2-year recurrence	209 (16.6)	125(12.4)	0.047	
Pelvic metastasis	131 (10.4)	78 (7.7)	0.028	
Distant metastasis	95 (7.5)	57 (5.6)	0.071	
5-year recurrence	282 (22.4)	167 (16.5)	<0.001	
Pelvic metastasis	180 (14.3)	98 (9.7)	<0.001	
Distant metastasis	139 (11.1)	78 (7.7)	0.007	
Bone	36 (2.9)	23 (2.3)	–	
Lung	57 (4.5)	23 (2.3)	–	
Liver	14 (1.1)	12 (1.2)	–	
Digestive tract	14 (1.1)	5 (0.5)	–	
Distant lymph nodes	34 (2.7)	9 (0.9)	–	
Others	9 (0.8)	12 (1.2)	–	
Side effects				
Short-term severe morbidity				
hematological toxicity				
Leucopenia	49 (3.9)	127 (12.6)	<0.001	
Neutropenia	12 (1.0)	164 (16.3)	<0.001	
Anemia	36 (2.9)	57 (5.6)	<0.001	
non-hematological toxicity				
Nausea/ Vomiting	131 (10.4)	190(18.8)	<0.001	
Diarrhea/bloody stools	242 (19.2)	69 (6.8)	<0.001	
AST/ALT	4(0.3)	31 (3.1)	<0.001	
Cystitis	69 (5.5)	5 (0.5)	<0.001	
Alopecie	23(1.8)	132 (13.1)	<0.001	
Dermal ulcer/Fistulous tract	51(4.1)	0(0.0)	<0.001	
Long-term severe morbidity				
Urinary complications	78 (6.2)	3 (0.3)	<0.001	
Bowel complications	125 (10.1)	5 (0.5)	<0.001	
Leg edema	65 (5.2)	13 (1.3)	<0.001	
Pelvis fibration	59 (4.7)	0(0.0)	<0.001	

Distant lymph nodes metastsis: Retroperitoneal lymph node, supraclavicular lymph nodes et al. AST, aspartate aminotransferase; ALT, alanine transaminase.

### Side Effects

In regards to short-term grade III and IV complications, severe hematological toxicity (neutropenia, 16.3% and leucopenia, 12.6%), nausea and vomiting (18.8%), and alopecia (13.1%) were more frequent in the chemotherapy group than the radiotherapy group (*p*<0.001; Table 1). Less frequent short-term complications included diarrhea (6.8%), anemia (5.6%), and abnormally increased levels of aspartate aminotransferase and alanine transaminase (3.1%). Radiotherapy-induced short-term severe toxicity mainly included diarrhea or bloody stools (19.2%), nausea and vomiting (10.4%), Cystitis (5.5%), dermal ulcer or fistulous tracts (4.1%), and leucopenia (3.9%).

Long-term severe morbidity after radiotherapy consisted mainly of bowel complications (10.1%), urinary complications (6.2%), leg edema (5.2%), and pelvis fibration (4.7%). There were fewer late severe side effects in the chemotherapy group compared with the radiation group.

### QoL and Sexual Activity Assessment

Less than one-fourth of the patients had hormone replacement therapy at the time of the QoL assessment, and no significant difference was found between the two groups (*p*>0.05; Table S4).

Significantly worse QoL outcomes were reported in several QoL domains after radiotherapy (150 patients) compared to chemotherapy (169 patients; Table 2). On the QLQ-C30 functioning scales, the radiotherapy group showed significantly lower mean scores for global health status/QoL, physical functioning, role functioning, and social functioning (*p*<0.05). On the symptom scales, patients in the radiotherapy group experienced significantly more symptoms (fatigue, nausea and vomiting, pain, and diarrhea) compared with patients in the chemotherapy group (*p*<0.05). Similarly, patients in the chemotherapy group had better QoL outcomes as evaluated by the EORTC QLQ-CX24 when compared to the radiotherapy group. Patients treated with radiotherapy had significantly lower scores on the functioning scales (body image and sexual/vaginal functioning) compared to patients in the chemotherapy group (*p* = 0.014 and *p* = 0.001, respectively), and experienced significantly more symptoms (symptom experience, lymphedema, and sexual worry: *p* = 0.003, *p* = 0.054, and *p* = 0.001, respectively). Patients in the radiotherapy group had significantly lower SAQ scores (sexual pleasure) compared to the chemotherapy group (*p*<0.001). Menopausal symptoms (i.e., hot flashes, vaginal dryness, and dyspareunia) were significantly increased after radiotherapy compared to chemotherapy (*p*<0.05).

**Table 2:** Comparison of Quality of Life, Sexual Functioning and Menopausal Symptoms in Radiotherapy and Chemotherapy Groups.

Scales (Items)	Radiotherapy group (*N = *150)	Chemotherapy group (*N* = 169)	*p* value	Effect size
EORTC QLQ-C30				
Global health status/QOL (29,30)	81.72	87.38	<0.001*	0.41
Physical functioning (1–5)	95.51	97.51	0.026*	0.28
Role functioning (6,7)	94.00	98.22	0.004*	0.40
Emotional functioning (21–24)	92.80	95.25	0.07*	0.22
Cognitive functioning (20,25)	98.11	98.31	0.798*	0.03
Social functioning (26,27)	92.84	97.11	0.005*	0.38
Fatigue (10,12,18)	5.04	1.92	0.013*	0.39
Nausea/Vomiting (14,15)	1.44	0.00	0.012*	0.41
Pain (9,19)	5.67	2.88	0.034*	0.29
Dyspnea (8)	2.44	0.60	0.082*	0.26
Insomnia (11)	10.00	11.24	0.606*	0.06
Appetite loss (13)	1.78	0.40	0.089*	0.25
Constipation (16)	4.88	5.95	0.545*	0.07
Diarrhea (17)	2.22	0.00	0.012*	0.42
Financial difficulties (28)	3.83	2.20	0.238*	0.14
EORTC QLQ-CX24				
Symptom experience (31–37, 39, 41–43)	2.75	1.65	0.003*	0.36
Body image (45–47)	95.36	98.32	0.014*	0.34
Sexual/Vaginal functioning (50–53)	83.52	88.46	0.001*	0.45
Lymphedema (38)	8.22	4.14	0.054*	0.24
Peripheral neuropathy (40)	3.11	3.63	0.684*	0.05
Menopausal symptoms (44)	8.39	6.34	0.330*	0.12
Sexual worry (48)	14.70	5.36	0.001*	0.51
Sexual activity (49)	30.90	35.36	0.102*	0.20
Sexual enjoyment (54)	32.42	36.22	0.178*	0.18
SAQ				
Sexual Pleasure (4–7,10,13)	7.18	8.95	<0.001*	0.42
Sexual Discomfort (8,9)	2.29	2.51	0. 329*	0.12
Sexual habits (12)	0.47	0.54	0.285*	0.13
Menopausal Symptoms				
**Hot flash**	**No. of Patients (%)**			
none/a little/moderate/a little much/ very much	120(81.1)/15(10.1) /4(2.7)/8(5.4)/1(0.7)	158(85.9)/18(9.8) /7(3.8)/1(0.5)/0(0.0)	0.039**	–
Urinary incontinence (when laugh or cry)				
none/a little/moderate/a little much/ very much	147(98.7)/1(0.7) /0(0.0)/1(0.7)/0(0.0)	165(99.4)/1(0.6) /0(0.0)/0(0.0)/0(0.0)	0.323**	–
Urinary incontinence(in other cases)				
none/a little/moderate/a little much/ very much	146(98.0)/2(1.3) /1(0.7)/0(0.0)/0(0.0)	164(98.8)/1(0.6) /1(0.6)/0(0.0)/0(0.0)	0.676**	–
Genital irritation / itching				
none/a little/moderate/a little much/ very much	138(93.9)/8(5.4) /1(0.7)/0(0.0)/0(0.0)	154(93.9)/8(4.9) /2(1.2)/0(0.0)/0(0.0)	0.869**	–
Vaginal dryness				
none/a little/moderate/a little much/ very much	85(65.4)/33(25.4) /7(5.4)/3(2.3)/2(1.5)	116(80.6)/18(12.5) /10(6.9)/0(0.0)/0(0.0)	0.008**	–
Dyspareunia				
none/a little/moderate/a little much/ very much	88(71.5)/27(22.0) /5(4.1)/2(1.6)/1(0.8)	124(87.3)/17(12.0) /0(0.0)/1(0.7)/0(0.0)	<0.001**	–
Night sweat				
none/a little/moderate/a little much/ very much	126(85.1)/11(7.4) /5(3.4)/5(3.4)/1(0.7)	144(86.7)/15(9.0) /7(4.2)/0(0.0)/0(0.0)	0.172**	–

* The P value is based on the Mann-Whitney nonparametric test for continuous variables.

** The P value is based on contingency-table analysis for categorical variables.

## Discussion

Radiotherapy greatly improves the survival of patients with cervical cancer; however, this treatment can cause permanent damage to pelvic organs, including functional damage in women at reproductive ages [12]. In the past 20 years, the morbidity has increased, as has the proportion of patients diagnosed at younger ages and early disease stages [2]. Thus, a comparable and appropriate treatment with the same curative effects and fewer complications is needed for these women of reproductive age. Gynecological oncologists have tried to treat cervical cancer with chemotherapy since the 1960s, but most chemotherapy regimens did not meet the treatment requirements due to short of ideal chemical reagents. In 1978, cisplatin was approved for clinical application by the U.S. Food and Drug Administration [28], after which two other new reagents, taxol and topotecan, were clinically approved [29], [30]. These three innovative drugs brought a new vitality to the treatment of cervical cancer. In the past decade, the clinical application of chemotherapy for cervical cancer has been extensively developed, and regimens that produce reliable curative effects, better control of distant metastasis, and less damage to the genital system have been developed.

In the present study, we collected data from 10,897 eligible cervical cancer cases (inpatients from 2002 to 2008) to compare the benefits and deficiencies of chemotherapy and radiotherapy. All of the chemotherapy regimens in the current study were cisplatin-based combinations, and approximately 50% consisted of taxol or topotecan (Table S2). An analysis of the data from all eligible cases revealed that patients who received chemotherapy did not have better 5-year long-term outcomes for chemotherapy than patients who received radiotherapy. However, a stratified analysis showed that chemotherapy was more effective than radiotherapy for selected patients who had early-stage disease, were younger in age, and exhibited clinical responses, suggesting that chemotherapy modalities provided preferable long-term outcomes for these patients. Moreover, the outcomes of patients who received current cisplatin-based chemotherapy modalities in this study were similar to the outcomes reported after concurrent chemoradiotherapy in several studies (Table S5) [20], [31]–[35].

Generally, metastasis, especially distant metastasis, is a major cause of death in patients with cervical cancer. Adjuvant chemotherapy is considered the most powerful means of eradicating subclinical distant metastases [19], [36]. However, the major concern with using chemotherapy alone is that local recurrence may increase in the absence of radiotherapy. Some patients who underwent primary radical hysterectomy were found to have HRFs for local recurrence, including LNM and parametrial invasion [37]–[39]. The data in the present study did not show any differences in the 5-year OS or DFS rates between the chemotherapy and radiotherapy groups (Fig. S2), indicating that the current cisplatin-based chemotherapy does not increase local recurrence or decrease the 5-year DFS rate. However, our statistical analysis clearly showed that distant metastasis and recurrence rates were reduced in the patients who received chemotherapy.

In addition to the prognosis of cervical cancer, another important issue to address is how to best preserve the function of genital organs for women of reproductive age without increasing the risk of recurrence. Irreversible side effects and QoL considerations become essential concerns when multiple treatment options with acceptable clinical outcomes are available to a patient. The present results indicated that patients who received pelvic region radiotherapy exhibited an increased incidence of long-term complications, including urinary disturbance, bowel obstruction, and pelvis fibration, which led to a worse overall QoL for survivors. It is generally accepted that chemotherapy has the advantage of causing less damage to genital organs, such as the ovaries and vagina. Furthermore, if necessary, even the uterus and reproductive ability can be preserved in patients receiving chemotherapy [21], [40].

Traditionally, chemotherapy has only been an option for recurrent cervical cancer or as an auxiliary treatment to enhance sensitivity to radiotherapy in concurrent chemoradiotherapy [3], [4]. However, we observed that the use of chemotherapy as a primary treatment in patients with early-stage disease, clinical responses, and younger ages resulted in increased survival rates. Moreover, compared with the non-reversible damage to pelvic organs caused by radiotherapy, the fewer side effects and better QoL in patients who received chemotherapy leads us to recommend chemotherapy as a conventional treatment for cervical cancer. Additionally, the retrospective analysis has limitations and that a prospective study of adjuvant chemotherapy is necessary to further confirm the results in this study.

## Supporting Information

Figure S1 **Patient enrollment in the present study.** (Note: The treatment modality not mentioned in this study was mainly including radiotherapy alone, radical surgery combined with adjuvant chemotherapy and radiotherapy, and concurrent chemoradiotherapy).
(TIF)

Figure S2 **Overall survival (OS) and disease-free survival (DFS) of patients with high-risk factors (HRFs) in the chemotherapy (CT) and radiotherapy (RT) groups.** Panels A and E show all patients with HRFs (187 CT and 172 RT). Panels B and F show patients with HRFs in stage IB (81 CT and 102 RT). Panels C and G show patients with HRFs in stage IIA (45 CT and 45 RT). Panels D and F show patients with HRFs in stage IIB-IIIB (61 CT and 25 RT). Panels I and K show patients with HRFs in stage IB1 (36 CT and 72 RT) and Panels J and L show patients with HRFs in stage IB2 (45 CT and 26 RT).
(TIF)

Table S1 **Chemotherapy Regimens in this Study.**
(DOC)

Table S2 **Radiotherapy Regimens in this Study.**
(DOC)

Table S3 **The Clinical and Pathological Characteristics in Radiotherapy and Chemotherapy Groups.**
(DOC)

Table S4 **Patient Characteristics in Radiotherapy and Chemotherapy Groups for the Quality of Life and Sexual Activity Assessment.**
(DOC)

Table S5 **Series of Adjuvant Treatments for Cervical Cancer.**
(DOC)
