# Supplementary figures and images for: Adjuvant Chemotherapy, a Valuable Alternative Option in Selected Patients with Cervical Cancer
Source: PLoS One. 2013 Sep 13;8(9):e73837. doi: 10.1371/journal.pone.0073837 (PMC3772826; doi:10.1371/journal.pone.0073837)

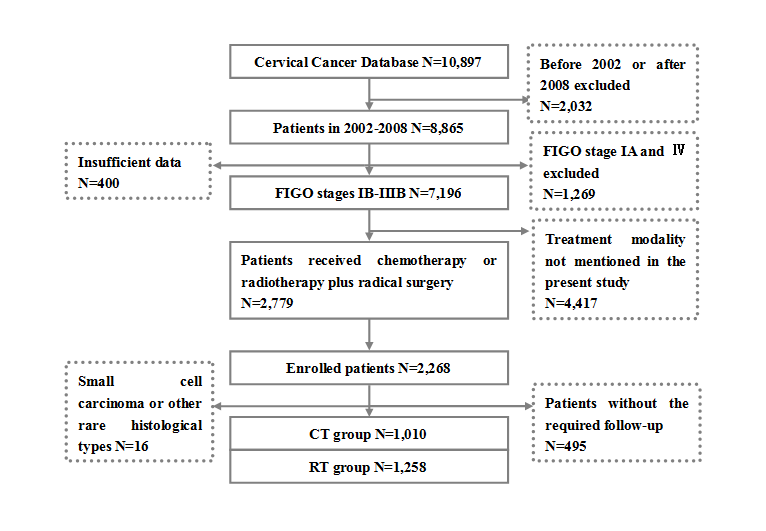

Supplement: Figure S1 — Patient enrollment in the present study. (Note: The treatment modality not mentioned in this study was mainly including radiotherapy alone, radical surgery combined with adjuvant chemotherapy and radiotherapy, and concurrent chemoradiotherapy). (TIF) [file pone.0073837.s001.tif]

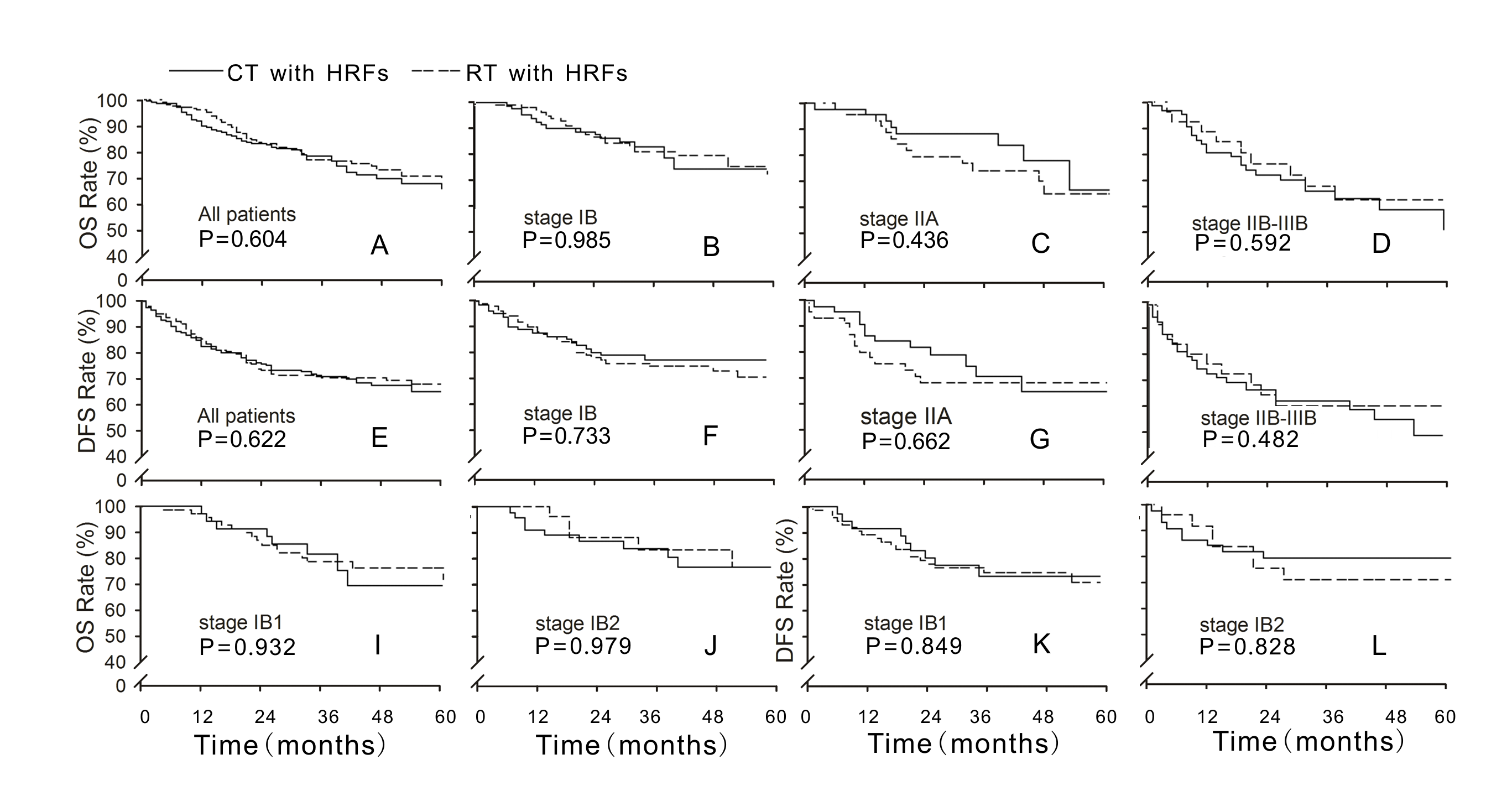

Supplement: Figure S2 — Overall survival (OS) and disease-free survival (DFS) of patients with high-risk factors (HRFs) in the chemotherapy (CT) and radiotherapy (RT) groups. Panels A and E show all patients with HRFs (187 CT and 172 RT). Panels B and F show patients with HRFs in stage IB (81 CT and 102 RT). Panels C and G show patients with HRFs in stage IIA (45 CT and 45 RT). Panels D and F show patients with HRFs in stage IIB-IIIB (61 CT and 25 RT). Panels I and K show patients with HRFs in stage IB1 (36 CT and 72 RT) and Panels J and L show patients with HRFs in stage IB2 (45 CT and 26 RT). (TIF) [file pone.0073837.s002.tif]
