# Supplementary material for: Adjuvant Chemotherapy, a Valuable Alternative Option in Selected Patients with Cervical Cancer
Source: PLoS One. 2013 Sep 13;8(9):e73837. doi: 10.1371/journal.pone.0073837 (PMC3772826; doi:10.1371/journal.pone.0073837)
Supplement: Table S1 — Chemotherapy Regimens in this Study. (DOC) [file pone.0073837.s003.doc]

| Table S1  Chemotherapy Regimens in this Study | | | |
| --- | --- | --- | --- |
| Schemes | Before Surgery | After Surgery | Chemotherapy Regimens |
|  | (*N* = 611) | (*N* = 664) |  |
|  | *no. of patients (%)* | |  |
| TP | 241 (39.4) | 286 (43.1) | Paclitaxel (day 1; 135-175 mg/m^2^; IV) and cisplatin (days 2-3; 80-85 mg/m^2^; IV). This treatment was repeated every 28 days. |
| BVP | 133 (21.8) | 93 (14.0) | Blemycin (days 1-3; 20 mg/m^2^/day; IV), VCR (day 2; 1 mg/m^2^; IV) and cisplatin (day 1; 75 mg/m^2^; IV). Two cycles were given at 14-day intervals. |
| PF | 57 (9.3) | 75 (11.3) | Cisplatin (day 1; 75 mg/m^2^; IV) and 5-Fu (days 1-5; 24 mg/kg/d; IV). This treatment was repeated at 3-week intervals. |
| BP | 37 (6.1) | 24 (3.6) | Blemycin (days 1-3; 20 mg/m^2^/day; IV), cisplatin (day 1; 75 mg/m^2^; IV). Two cycles were given at 14-day intervals. |
| PFM | 30 (4.9) | 18 (2.7) | Cisplatin (day 1; 100 mg/m^2^; IV), mitomycin C (days 1-5; 4 mg/m^2^; IM) and 5-fluorouracil (days 1-5; 24 mg/kg/day; IV). Two cycles were given at 14-day intervals. |
| CP | 35 (5.7) | 61 (9.2) | Irinotecan hydrochloride (days 1, 8, and 15; 60 mg/m^2^; IV), and cisplatin (day 1; 60 mg/m^2^; IV). This treatment was repeated every 28 days. |
| Others* and unknown | 78 (12.8) | 107 (16.1) |  |
| IV: intravenation; IM: intramuscular injection;  *Other regimens include BIP (blemycin, iphosphamide, and cisplatin), BEP (blemycin, pharmorubicin, and cisplatin), PAF (cisplatin, adriamycin and 5-FU), and PIF (cisplatin, iphosphamide, and 5-FU) and so on. | | | |
