# Supplementary material for: Adjuvant Chemotherapy, a Valuable Alternative Option in Selected Patients with Cervical Cancer
Source: PLoS One. 2013 Sep 13;8(9):e73837. doi: 10.1371/journal.pone.0073837 (PMC3772826; doi:10.1371/journal.pone.0073837)
Supplement: Table S2 — Radiotherapy Regimens in this Study. (DOC) [file pone.0073837.s004.doc]

| Table S2  Radiotherapy Regimens in this Study | | | |
| --- | --- | --- | --- |
| Schemes | Before Surgery | After Surgery | Radiotherapy Regimens |
|  | (*N* = 986) | (*N* = 370) |  |
|  | *no. of patients (%)* | |  |
| Intracavitary brachytherapy | 952 (96.6) | 50 (13.5) | Pre-RT group: mean total dose 22.07 Gy; Range 5-90 Gy;  Post-RT group: mean total dose 22.92 Gy; Range 5-61 Gy. |
| External beam irradiation | 31 (3.1) | 211 (57.0) | Pre-RT group: mean total dose: 30.39 Gy; Range 4-90 Gy;  Post-RT group: mean total dose: 42.93 Gy; Range 2-92 Gy; |
| Others and unknown | 17 (1.7) | 142 (38.4) |  |
