# Supplementary material for: Adjuvant Chemotherapy, a Valuable Alternative Option in Selected Patients with Cervical Cancer
Source: PLoS One. 2013 Sep 13;8(9):e73837. doi: 10.1371/journal.pone.0073837 (PMC3772826; doi:10.1371/journal.pone.0073837)
Supplement: Table S3 — The Clinical and Pathological Characteristics in Radiotherapy and Chemotherapy Groups. (DOC) [file pone.0073837.s005.doc]

| Table S3  The Clinical and Pathological Characteristics in Radiotherapy and Chemotherapy Groups | | | |
| --- | --- | --- | --- |
|  | Radiotherapy Group | Chemotherapy Group | *p* value |
|  | (*N* = 1,258) | (*N* = 1,010) |
|  | *no. of patients (%)* | |  |
| Age (years) | | | |
| ≤40 | 469 (37.3) | 418 (41.4) | 0.058 |
| >40 | 780 (62.0) | 590 (58.4) |  |
| Unknown | 9 (0.7) | 2 (0.2) |  |
| Married status | | | |
| Yes | 1,240 (98.6) | 987 (97.7) | 0.132 |
| No | 17 (1.4) | 22 (2.2) |  |
| Unknown | 1 (0.1) | 1 (0.1) |  |
| FIGO stage | | | |
| IB-IIA | 1,179 (93.7) | 799 (79.1) | <0.001 |
| IIB-IIIB | 77 (6.1) | 205 (20.3) |  |
| Unknown | 2 (0.2) | 6 (0.6) |  |
| Histology | | | |
| SqCC | 1,214 (96.5) | 872 (86.3) | <0.001 |
| AC/ASC | 41 (3.3) | 133 (13.2) |  |
| Unknown | 3 (0.2) | 5 (0.5) |  |
| Tumor size | | | |
| ≤ 4 cm | 909 (72.3) | 519 (51.4) | <0.001 |
| > 4 cm | 332 (26.4) | 472 (46.7) |  |
| Unknown | 17 (1.4) | 19 (1.9) |  |
