# Supplementary material for: Adjuvant Chemotherapy, a Valuable Alternative Option in Selected Patients with Cervical Cancer
Source: PLoS One. 2013 Sep 13;8(9):e73837. doi: 10.1371/journal.pone.0073837 (PMC3772826; doi:10.1371/journal.pone.0073837)
Supplement: Table S4 — Patient Characteristics in Radiotherapy and Chemotherapy Groups for the Quality of Life and Sexual Activity Assessment. (DOC) [file pone.0073837.s006.doc]

| Table S4  Patient Characteristics in Radiotherapy and Chemotherapy Groups for the Quality of Life and Sexual Activity Assessment. | | | | |
| --- | --- | --- | --- | --- |
|  | Radiotherapy Group  (*N* = 102) | | Chemotherapy Group  ( *N* = 128) | *p* value |
|  | *no. of patients* (%) | | |  |
| Age | | | | |
| mean, SD | | 38.7 ± 5.3 | 37.4 ± 5.3 | 0.077 |
| ≤ 30 | | 9 (8.8) | 13 (10.2) | 0.938 |
| 30-40 | | 60 (58.8) | 75 (58.6) |  |
| 40-50 | | 33 (32.4) | 40 (31.3) |  |
| Unknown | | 0 (0.0) | 0 (0.0) |  |
| Married status | | | | |
| Yes | | 95 (93.1) | 114 (89.1) | 0.705 |
| No | | 4 (3.9) | 3 (2.3) |  |
| Unknown | | 3 (2.9) | 11 (8.6) |  |
| Region | | | | |
| Urban | | 60 (58.8) | 52 (40.6) | 0.062 |
| Rural | | 28 (27.5) | 43 (33.6) |  |
| Unknown | | 14 (13.7) | 33 (25.8) |  |
