# Supplementary material for: Adjuvant Chemotherapy, a Valuable Alternative Option in Selected Patients with Cervical Cancer
Source: PLoS One. 2013 Sep 13;8(9):e73837. doi: 10.1371/journal.pone.0073837 (PMC3772826; doi:10.1371/journal.pone.0073837)
Supplement: Table S5 — Series of Adjuvant Treatments for Cervical Cancer. (DOC) [file pone.0073837.s007.doc]

| Table S5  Series of Adjuvant Treatments for Cervical Cancer | | | | | | | | | | | | |
| --- | --- | --- | --- | --- | --- | --- | --- | --- | --- | --- | --- | --- |
| Authors | Stage | Numbers | Treatment | OS rate (%) | *p* value | DFS rate (%) | *p* value | LRR (%) | *p* value | DMR (%) | *p* value | Publishment |
| Mitchell M, et al. (1999) ^26^ | IB-IVA (5Y） | 147/193 | CCRT/RT | 73.0/58.0 | 0.004 | 67.0/40.0 | <0.001 | 19.0/35.0 | <0.001 | 14.0/33.0 | <0.001 | New Engl J Med |
| William A, et al. (2000) ^15^ | IB-IIA (4Y) | 106/127 | CCRT/RT | 81.0/71.0 | 0.007 | 80.0/63.0 | 0.003 | 9.0/22.0 | 0.020 | 10.0/22.0 | 0.020 | J Clin Oncol |
| Kiyosumi Shibata, et al. (2008) ^27^ | IB-IIB (5Y) | 37/52 | CCRT/RT | 91.9/75.0 | 0.087 | 89.2/69.2 | 0.039 | -/- |  | -/- |  | Int J Clin Oncol |
| Hideomi Yamashita, et al.(2010) ^28^ | IIB (3Y) | 34/25 | CCRT/ RT | 75.0/80.0 | NS | 79.0/70.0 | NS | 16.0/12.0 | NS | 4.0/18.0 | NS | Am J Clin Oncol |
| The current study | IB-III (5Y) | 1010/1258 | CT/RT | 86.5/82.8 | 0.053 | 84.5/81.4 | 0.093 | 9.7/14.3 | <0.001 | 7.7/11.1 | 0.007 |  |
| Abbreviation: OS: overall survival; DFS: disease-free survival; LRR: local (vagina or pelvis) recurrence rate; DMR: distant metastasis rate; NS: no significance | | | | | | | | | | | | |
